# Up-Regulation of Antioxidant Proteins in the Plasma Proteome during Saturation Diving: Unique Coincidence under Hypobaric Hypoxia

**DOI:** 10.1371/journal.pone.0163804

**Published:** 2016-10-14

**Authors:** Hideharu Domoto, Keiichi Iwaya, Fumitaka Ikomi, Hirotaka Matsuo, Yutaka Tadano, Shigenori Fujii, Kazuyoshi Tachi, Yoshiyuki Itoh, Michiya Sato, Kimitoshi Inoue, Nariyoshi Shinomiya

**Affiliations:** 1 Research Division, Maritime Self-Defense Force Undersea Medical Center, Yokosuka, Kanagawa, Japan; 2 Department of Pathology, SASAKI Institute, Kyoundo Hospital, Chiyoda, Tokyo, Japan; 3 National Defense Medical College Research Institute, Tokorozawa, Saitama, Japan; 4 Department of Integrative Physiology and Bio-Nano Medicine, National Defense Medical College, Tokorozawa, Saitama, Japan; 5 JEOL Ltd., Akishima, Tokyo, Japan; Hokkaido Daigaku, JAPAN

## Abstract

Saturation diving (SD) is one of the safest techniques for tolerating hyperbaric conditions for long durations. However, the changes in the human plasma protein profile that occur during SD are unknown. To identify differential protein expression during or after SD, 65 blood samples from 15 healthy Japanese men trained in SD were analyzed by two-dimensional fluorescence difference gel electrophoresis. The expression of two proteins, one 32.4 kDa with an isoelectric point (pI) of 5.8 and the other 44.8 kDa with pI 4.0, were elevated during SD to 60, 100, and 200 meters sea water (msw). The expression of these proteins returned to pre-diving level when the SD training was completed. The two proteins were identified using in-gel digestion and mass spectrometric analysis; the 32.4 kDa protein was transthyretin and the 44.8 kDa protein was alpha-1-acid glycoprotein 1. Oxidation was detected at methionine 13 of transthyretin and at methionine 129 of alpha-1-acid glycoprotein 1 by tandem mass spectrometry. Moreover, haptoglobin was up-regulated during the decompression phase of 200 msw. These plasma proteins up-regulated during SD have a common function as anti-oxidants. This suggests that by coordinating their biological effects, these proteins activate a defense mechanism to counteract the effects of hyperbaric-hyperoxic conditions during SD.

## Introduction

Saturation diving (SD) is a diving technique that allows divers to be safe underwater and under high pressure for long durations [1,2]. Generally, SD seems to be an under-recognized and very specialized diving technique. However, for some commercial companies and military, SD is a requisite for accomplishing their missions. The usefulness of SD has also been recognized by underwater laboratories studying coral biology and reef ecology. In addition, SD can be used as a fundamental tool to study human adaptation to high pressure, contributing to our knowledge of diving medicine and physiology and to hyperbaric and hypobaric medicines. Under ambient pressure (normobaric normoxia), our body is saturated with nitrogen, an inert gas. Therefore, altitude decompression sickness may occur with exposure to altitude changes in a flight chamber or sudden loss of cabin pressure in an aircraft [1]. SCUBA diving is a very well-known and convenient diving method, but the time a diver can stay in the water (bottom duration) is limited by gas supply [1]. In addition, bottom duration (in-water working time) generally decreases with dive depth due to the time required to exhale the dissolved gas from the body during decompression to the surface [1].

Diving efficiency (DE) is calculated by the following equation: DE = bottom duration (in-water working time) / dive duration (total time of diving). SD enables humans to survive under hyperbaric conditions at depths greater than 100 meters sea water (msw) for long durations [1,2]. During SD, various organs of the human body absorb the maximum partial pressure of helium (an inert gas) due to exposure to hyperbaric conditions for prolonged periods. Once human tissues become saturated, the time to ascend to the surface will not increase with further exposure. Therefore, the longer we conduct SD, the more DE increases. Thus, good DE can be achieved by SD. To conduct SD, deck decompression chambers (habitat for divers), a personnel transfer capsule (in-water elevator), and various kinds of supporting machines (circulator, air conditioner, CO_2_ scrubber, etc.) are required to maintain a safe environment for divers, contributing to a high running cost and a need for man power as well. In Japan, only the Japan Maritime Self-Defense Force (JMSDF) and Ocean Works Asia Inc. (Tokyo) presently have SD capability. Generally, SD has been recognized as very safe and suitable for prolonged underwater work such as construction of an underwater platform [1,3,4,5], where skilled human hands are required rather than powerful robots [1,6].

JMSDF Undersea Medical Center (Yokosuka, Kanagawa, Japan) has established an SD system in Japan where the linear decompression protocol is used as a template [7,8]. Since 1977, many divers have been involved in SD, mainly for the purpose of emergency submarine rescue operations. JMSDF SD profiles maintain the partial pressure of oxygen at 0.42 atmospheric absolute (ATA) from the point of compression to the beginning of decompression and 0.5 ATA from the rest of decompression to the point of reaching the surface [8].

During SD with storage depths greater than 150 msw, various physiological and pathological conditions occur in the human body, primarily during compression to the bottom phase. These include high pressure nervous syndrome [9], hyperbaric bradycardia [10], hyperbaric diuresis [11], and hyperbaric arthralgia [12]. During the decompression phase, decompression sickness might occur, although the overall risk should be lower for SD than for other diving techniques. Among the long term health effects on divers involved in frequent SD are harmful neurological effects that have been previously proposed by some authors [13,14]; however, a recent publication described SD as being reasonably safe and well controlled, with no documented long-term health impact [15,16]. To our knowledge, we have never encountered neurological deficits among saturation divers from the JMSDF. SD does induce various physiological and biochemical changes in the immune system [17–19] and blood cell count [20,21], and increases the levels of reactive oxygen species [15,22], heat shock proteins [15,18,23], antioxidants [15,22,24], nitric oxide [23], and cytokines. Of these and other changes, cytotoxicity of natural killer cells [19], lymphocyte subset change [17,18], and expression of heart shock proteins [18] have been described as possible candidates for biological stress markers in divers during SD.

SD exposes divers to hyperbaric hyperoxia, and the resultant stress reaction is likely to activate the antioxidant system. The interrelationship between hyperoxia and free radicals has been previously described to explain oxygen toxicity [25] and vascular endothelial function [26]. Hypobaric hypoxia, which is the diametric opposite of hyperbaric hypoxia, is also known to increase the generation of reactive oxygen species and antioxidants similar to hyperbaric hyperoxia. The proposed mechanism is that the relative oxygen insufficiency limits the availability of oxygen for its reduction to H_2_O by cytochrome oxidase [27], leading to the accumulation of superoxide radicals in the mitochondrial respiratory chain, also known as reductive stress [27]. Recent advancements in proteomic analysis clearly showed the increased expression of antioxidant proteins after exposure to hypobaric hypoxia [27–30].

In this study, two-dimensional fluorescence difference gel electrophoresis (2D DIGE) and mass spectrometric analysis were used to analyze the blood samples of saturation divers from JMSDF to detect major changes in the profile of plasma proteins. We also proposed possible biomarkers for studying the effects of SD.

## Materials and Methods

### Subjects

SD trainings were conducted for 15 divers at the JMSDF Undersea Medical Center from 2012 to 2013. These divers were divided into three groups and were exposed to different levels of helium-oxygen atmosphere: 60 msw (n = 4), 100 msw (n = 5), or 200 msw (n = 6). We reached 450 msw in the deep diving simulator at JMSDF Undersea Medical Center and at the sea by using SD techniques. Because of budgetary constraints and limited human resources needed to conduct SD safely, SD is usually performed a limited number of times in a year for research and training purposes, with storage depths varying between 100 and 450 msw depending on the year. In addition, 60 msw SD is regularly conducted 2–3 times in a year as part of the final phase of the “saturation divers course,” originally established by JMSDF. Therefore, all JMSDF saturation divers have experienced 60 msw saturation dives at the Undersea Medical Center before they become certified.

**Fig 1:**
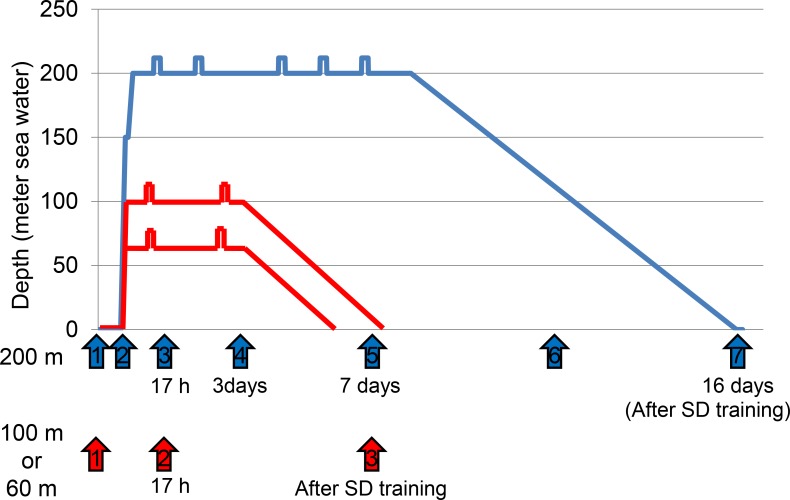
Schedule of blood sampling during saturation diving (SD) at 60, 100, and 200 msw. At 60 msw (n = 4) and 100-msw (n = 5) SD, venous blood was sampled from 9 divers at three time points: just before SD training, 17 h after reaching the respective target depths, and just after SD training. The time points are shown by red arrows (1–3). In 200 msw SD, venous blood was sampled at the same three time points mentioned previously plus 4 additional time points (compression phase reached at 100 msw, day 3 and day 7 in the hyperbaric condition at 200 msw, and the decompression phase at 100 msw). The 7 time points are indicated by blue arrows (1–7) during SD to 200 msw in 5 of 6 divers.

All divers were healthy Japanese men who were expert undersea divers, and none of them had ever experienced decompression sickness. The time schedules for blood sampling are shown in Fig 1. From the 9 divers who participated in SD training at either 60 msw or 100 msw, venous blood was sampled at three time points: (1) just before and (2) after SD training, and (3) after 17 h exposure to hyperbaric conditions (at 60 msw or 100 msw). From the 6 divers who were trained at 200 msw, blood was sampled at 7 time points: (1) just before SD training, (2) when the compression phase reached 100 msw, at (3) 17 h, (4) 3 days, or (5) 7 days in the hyperbaric condition at 200 msw, (6) when the decompression phase reached 100 msw, and (7) just after returning to normal pressure. All divers in this group underwent blood sampling at the scheduled 7 time points with the exception of one diver, who missed the blood sampling at 4 time points (at 17 h, 3 days, and 7 days in the hyperbaric condition at 200 msw, and when the decompression phase reached 100 msw). In total, 65 venous blood samples were collected in EDTA-treated vials, kept for 30 min at about 37°C, and centrifuged at 1500 ×*g* for 10 min. Supernatants were transferred to new tubes in aliquots of 250 μL. The plasma samples were stored at -80°C until assayed. All samples were processed within 1 h of collection and no hemolysis was detected. Plasma samples were titrated with 1:10 (v/v) cold acetone, precipitated for 2 h at -20°C, and centrifuged at 12,000 ×*g* for 30 min at 4°C. The supernatants were discarded and the plasma precipitates were dissolved in the rehydration buffer (GE Healthcare Japan, Hino, Japan) and stored at -80°C. Written informed consent was obtained from all 15 divers; procedures conformed to the declaration of Helsinki. This study was approved by the ethics committees of the Japan Maritime Self-Defense Force and the National Defense Medical College.

### 2D DIGE

Proteins were labeled with fluorescent cyanine dyes (GE Healthcare Japan) in accordance with the manufacturer's instructions. In brief, 50 μg of a pair of samples were adjusted to pH 8.5 with 50 mM NaOH and labeled with 400 pmol Cy3 or Cy5, whereas another pair of samples were labeled inversely with Cy5 or Cy3. The fluorescence labeling was performed on ice in the dark for 30 min, and then quenched with 1 μl of 10 mM lysine (Sigma-Aldrich, St. Louis, USA) for 10 min. Each preparation was treated with 2X sample buffer containing 7 M urea, 2 M thiourea, 4% CHAPS, 1% immobilized pH gradient (IPG)-buffer, pH range 4–7, and 2% dithiothreitol (DTT). The final volume was adjusted to 260 μl with rehydration buffer (7 M urea, 2 M thiourea, 4% CHAPS, 0.5% IPG-buffer, pH range 4–7, 0.2% DTT). The proteins were applied to Immobiline DryStrips (pH 4–7, 13 cm) and focused on an Ettan IPGphor II (GE Healthcare Japan).

Four focused IPG strips were equilibrated and then loaded onto 12.5% SDS–polyacrylamide gels (30% acrylamide, 1.5 M Tris-HCl pH 8.8, 10% SDS, 10% APS, 10% TEMED) using low-fluorescence glass plates on an Ettan DALT II system (GE Healthcare Japan). All electrophoresis procedures were performed in the dark. After SDS–polyacrylamide gel electrophoresis, pairs of gels were scanned with a Typhoon 9410 scanner (GE Healthcare Japan) at appropriate excitation/emission wavelengths specific for Cy3 (532/580 nm) and Cy5 (633/670 nm) to generate protein spot maps. The above procedure was repeated three times. DeCyder 7.0 software (GE Healthcare Japan) was used for 2D DIGE analysis in accordance with the manufacturer's recommendation. The DeCyder differential in-gel analysis module was used for pairwise comparisons of each sample. Differential protein spots (ratio >2, *P* < 0.05) that showed consistent alteration were selected for further analysis.

### In-gel digestion and mass spectrometric analysis

Peptide mass fingerprinting (PMF) was performed for mass spectrometric analysis. To extract the differential protein spots, 500 μg of unlabeled sample was loaded on 2D SDS gel as described above. Separated spots on the gel were then visualized with modified Coomassie blue staining (Quick-CBB^®^, Wako Pure Chemical Industries Ltd., Osaka, Japan) for 2 h. After three washes (10 min each) with distilled water, each spot was excised from the gel using a 1 mL pipet and placed in a PCR plate with water. In-gel digestion was performed as follows. Briefly, reduction was done using 15 mg DTT in 50 mM ammonium bicarbonate (ABM) and alkylation was done using 20 mg iodoacetamide in 50 mM ABM. The gel plugs were washed in an ultrasonic water bath with ice for 10 min, dehydrated with 100% acetonitrile (ACN), and incubated in vacuo for 10 min. Each procedure was repeated 4–6 times. After drying for 1 h, the gel plugs were digested overnight in 0.5% trypsin in 50 mM ABM. The trypsin digests were recovered with 70% ACN and 0.1% trifluoroacetic acid (TFA). The gel plugs were then subjected to speedvac at 15°C for 20 min. Finally, 15 μl of 0.1% TFA was added.

The trypsin digests were subjected to both spiral mass spectrometry (JMS-S3000 MALDI-spiral TOF-TOF, Japan Electron Optics Laboratory, Akishima, Japan) and liquid chromatography-mass spectrometry (AB SCIEX, Framingham, MA, USA). A database search was conducted using the Mascot search engine at www.matrix-science.com. Proteins that scored greater than 62 were considered significant (*P* < 0.05).

### 2D western blot analysis

After the 2D gel electrophoresis described above, separated protein spots were blotted onto PVDF membranes (Amersham Hybond P PVDF 0.45, GE Healthcare Japan). Transthyretin (TTR) was detected with an anti-human TTR mouse monoclonal antibody (Proteintech, Chicago, IL, USA) diluted to 1:10,000 and was visualized using chemiluminescence (ECL Plus, GE Healthcare Japan). Signals were detected using the LAS3000 image analyzer (Fuji film, Kanagawa, Japan) and DeCyder ver. 7.0 (GE healthcare Japan). To confirm the position of each spot, blotted membranes were stained with gold-colloid stain (GE Healthcare Japan).

## Results

### 2D DIGE

**Fig 2:**
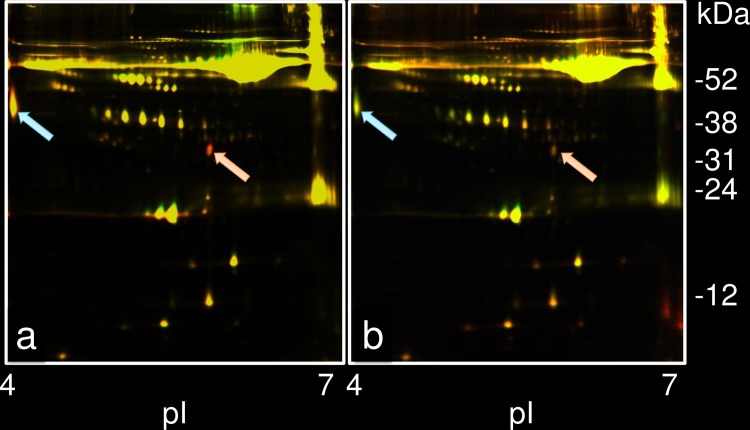
A representative result of 2D DIGE for a diver diving at 60 msw. (a) Differential expression of two plasma protein spots is shown for the hyperbaric condition at 60 msw for 17 h compared with that before SD training in a healthy diver. The red spot, designated as the increased expression spot, was detected at 32.4 kDa, pI 5.8 (ratio; 2.33), and indicated by a pink arrow. The other reddish spot indicated by a light blue arrow was located at 44.8 kDa, pI 4.0 (ratio; 1.85). (b) Differential expression of plasma protein spots pre- and post-SD training from the same diver. The 32.4-kDa spot decreased to about the same level (ratio; 1.21) (indicated by a pink arrow). The expression of the 44.8-kDa protein decreased to below pre-SD training level (ratio; -1.78), detected as a green spot (indicated by a light blue arrow).

To detect changes in the plasma protein profile under SD conditions, 2D DIGE was performed on the sera of 9 divers sampled at 17 h of exposure to hyperbaric conditions at 60 msw and/or 100 msw, and these results were compared with those before SD training. Fig 2A shows a case where the levels of two spots increased under SD at 60 msw, (indicated by arrows as red spots). One spot was located at 32.4 kDa with pI 5.8, and the other at 44.8 kDa with pI 4.0. Both the spots were analyzed using the DeCyder differential in-gel analysis module. To calculate the expression level, the protein spot volume (sum of pixel intensity within the spot boundary) of the corresponding spot before SD was considered as 1.00. The increase in the 32.4 kDa spot ranged from 2.09- to 2.55-fold with an average of 2.30-fold. This increase was significant in all 9 divers. The 44.8 kDa spot showed an increase that ranged from 1.54- to 4.37-fold; average of 1.98-fold. This increase was significant in only 2 of 9 divers.

The protein spot volumes of these two spots decreased when the SD training was completed. As seen from the differential profile of plasma proteins before and after SD training in Fig 2B, the spot at 32.4 kDa became small. The high intensity of this spot during hyperbaric conditions decreased to about the same pre-SD level of spot ratio (1.06 to 1.30; average, 1.20) in all 9 divers after completion of the training. For the 44.8 kDa spot, the expression during SD was lower than that during pre-SD training (spot ratio range: -1.86 to -1.76; average, -1.75).

No significant changes were detected in any other spot with the exception of the haptoglobin (Hp) spot, which showed higher expression during SD at 100 msw than at the normal levels as determined from the standard protein map of human plasma.

**Fig 3:**
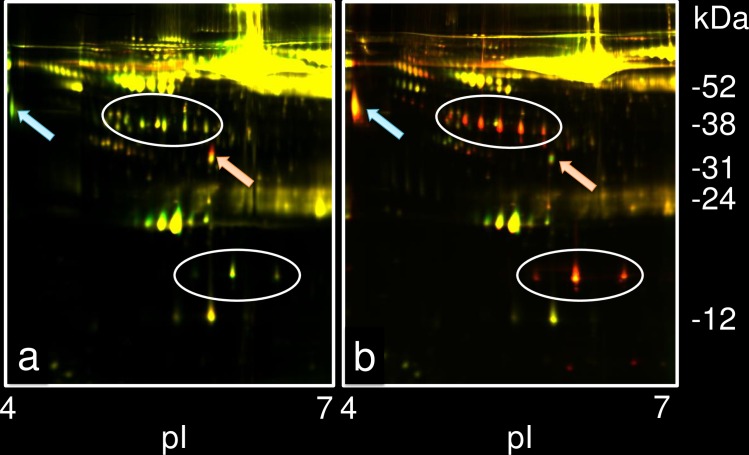
A representative result of 2D DIGE in a diver at 200 msw. (a) Differential expression of plasma proteins in hyperbaric conditions at 200 msw for 17 h compared with that before SD training in a healthy diver. The red spot indicated by a pink arrow was located at 32.4 kDa, pI 5.8 (ratio; 2.18). The green spot indicated by a light blue arrow was located at 44.8 kDa, pI 4.0 (ratio;-1.32). (b) Differential expression of plasma proteins during the decompression phase at 100 msw and pre-SD training in the same diver. The 32.4-kDa spot decreased to about the same level, indicated by a pink arrow (ratio; 1.25). The expression of the 44.8-kDa protein increased (ratio; 2.73), indicated by light blue arrow. High expression of Hp is indicated by the spots circled in white.

For SD at 200 msw, the 32.4 kDa protein spot volume significantly increased in all 6 divers at the 17 h exposure time point (range of 2.02–2.74; average of 2.21) and a representative case is shown in Fig 3A (increase to 2.18). The average spot volume at 100 msw of the compression phase slightly increased to 1.21 (range of 1.10–1.27). After 17 h exposure in the bottom phase at 200 msw, the spot volume of the 32.4 kDa spot remained high: 2.03 (range of 1.84–2.36) on day 3 and 2.37 (range of 2.11–2.87) on day 7. However, the spot volumes decreased rapidly to 1.11 (range of 1.09–1.25) in the decompression phase at 100 msw. Immediately after the completion of the SD training, the spot volume was further reduced to almost the pre-SD level (average of 1.03; range of -1.08–1.33).

In contrast, the expression of the 44.8 kDa protein changed variably during SD at 200 msw. The average spot volume of the 44.8-kDa protein when the compression phase reached 100 msw was 2.37 (range of 1.75–3.06). It decreased to 1.27 (range of -1.32–2.95) at 17 h of exposure to 200 msw SD (Fig 3A). During the final conditioning at 200 msw, the average spot volume increased to 1.94 (range of 1.26–2.44) on day 3 and decreased to -1.71 (range of -1.98–-1.33) on day 7. Although variable changes were detected in the 44.8 kDa protein during 200 msw SD, including the compression phase at 100 msw, significant up-regulation was detected in all 6 divers. The expression of the spot was high in the decompression phase at 100 msw (average of 3.45; range of 2.52–5.99; Fig 3B), and it decreased (average of 1.13; range, -1.30 to 1.67) when the training was completed.

Hp levels were significantly elevated during the decompression phase at 100 msw and immediately after the return to normal atmosphere in all 6 divers at 200 msw SD (Fig 3B).

### In-gel digestion and mass spectrometric analysis

The 32.4-kDa spot was identified as the A chain of normal human transthyretin (TTR) by Mascot search (*P* < 0.05) (Table 1A). All 127 amino acid residues of TTR were matched, and error tolerance research detected all significant protein hits. As for modifications, oxidation occurred at methionine 13. Because some of the 32.4-kDa spots were suspected to be composed of two spots, they were divided into two parts and the amino acid sequences were examined independently. However, no differences in the sequence data were detected between the two parts. The 44.8 kDa spot was identified as alpha-1-acid glycoprotein 1 (AGP) (*P* < 0.05) (Table 1B). Oxidation at methionine 129 was also detected.

**Table 1:** Matched protein sequences of serum proteins.

**(a) transthyretin**					
**1**	**GPTGTGESKC**	**PL****M****VKVLDAV**	**RGSPAINVAV**	**HVFRKAADDT**	**WEPFASGKTS**
**51**	**ESGELHGLTT**	**EEEFVEGIYK**	**VEIDTKSYWK**	**ALGISPFHEH**	**AEVVFTANDS**
**101**	**GPRRYTIAAL**	**LSPYSYSTTA**	**VVTNPKE**		
**(b) alpha-1-acid glycoprotein1**					
**1**	MALSWVLTVL	MALSWVLTVL	PLCANLVPVP	ITNATLDQIT	GK**WFYIASAF**
**51**	**R**NEEYNKSVQ	EIQATFFYFT	PNKTEDTIFL	REYQTRQDQC	IYNTTYLNVQ
**101**	R**ENGTISRYV**	**GGQEHFAHLL**	**ILRDTKTY****M****L**	**AFDVNDEKNW**	**GLSVYADKPE**
**151**	**TTKEQLGEFY**	**EALDCLRIPK**	**SDVVYTDWKK**	**DK**CEPLEKQH	EKERKQEEGE
**201**	S				

Matched peptides are shown in bold formatting.

Oxidized aminoacids are underlined.

### 2D western blot analysis

**Fig 4:**
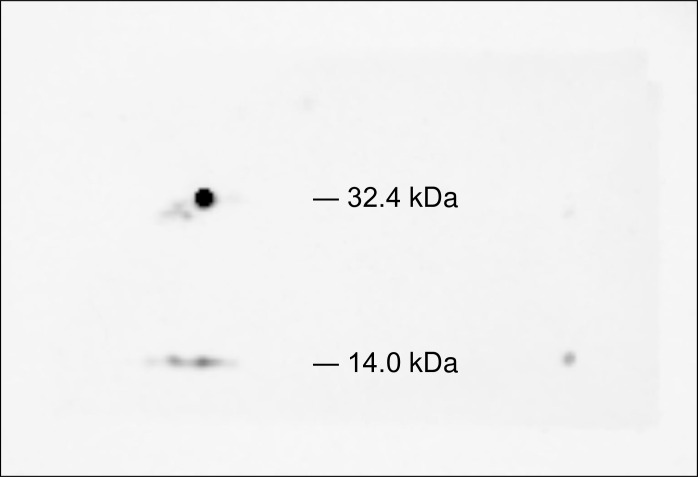
2D western blot analysis using anti-TTR antibody. The 32.4-kDa spot distinctly reacted with the anti-transthyretin antibody. Weak immunoreaction at 14.0 kDa was also detected and was interpreted as a monomer of TTR. The blotted membrane was stained with gold-colloid stain, and the area of immunoreaction was confirmed to correspond to the 32.4-kDa spot detected by 2D DIGE.

The data from the 2D DIGE indicated that the 32.4 kDa spot, 100% match with TTR identified by PMF, is the most promising candidate marker for SD. However, the expected molecular weight was 13,810 Da. To confirm the result of the Mascot search, 2D western blot analysis was performed using anti-TTR antibody. The 32.4 kDa spot showed distinct immunoreactivity with the anti-TTR antibody (Fig 4). Weak immunoreactivity at 14.0 kDa spot was also detected, which is assumed to be a monomer of TTR.

## Discussion

The present proteomic analysis is the first of its kind to investigate differential expression of proteins after exposure to hyperbaric hyperoxia during SD. We detected increased expression of TTR and AGP during SD at 60, 100, and 200 msw. In addition, up-regulation of Hp was identified from the bottom phase to the surface in 200 msw SD. The TTR expression was constantly high during the bottom phase and rapidly decreased during the decompression phase. In contrast, the AGP expression pattern during the bottom phase was inconsistent, and high expression was detected during the decompression phase. These data suggest that the TTR expression level is well correlated with the pressure and is a promising biomarker of hyperbaric conditions.

TTR is one of the abundant plasma proteins known as pre-albumins, which transports thyroxine and retinol binding protein. TTR is a known negative acute-phase protein, antioxidant protein, and an indicator of the nutritional status of patients [31]. Recently, the relationship between the dissociation of TTR’s tetrameric form to monomeric form and oxidative stress [32], and the multiple functions of TTR’s oxidative form have been reported [33–35]. Among them, the amyloidogenicity of TTR and its role in neuropsychological diseases appear to garner much attention [36–39]. To our knowledge, however, no relationship has been proposed between SD and neurological abnormalities due to the amyloidogenicity of TTR.

One unique feature of TTR detected by 2D DIGE in this study is its molecular weight (MW) at 32.4 kDa. Theoretically, the MW of monomeric TTR is 14 kDa, which was detected as a non-differential spot in our 2D DIGE. The in-gel digested 32.4-kDa protein was 100% match with human TTR by the Mascot search. Although the three-dimensional structure of TTR at 32.4 kDa is unknown, we suggest that conformational changes might have occurred since ELISA did not successfully detect the 32.4 kDa TTR (data not shown). We speculate that the oxidative modification of TTR might have affected its conformation or that the concentration of urea used in the denaturing procedure for 2D DIGE was not sufficient to release the native conformation of TTR.

AGP, one of the acute phase proteins, is also abundant in human plasma and is heavily glycosylated [40]. Recently, AGP has been correlated with malignancy, such as in pancreatic cancer and hepatocellular carcinoma [41,42]. The glycosylation pattern of AGP is different in different diseases. The concentration of AGP increases several fold during acute events such as severe burns or trauma, as well as under chronic pathological conditions like rheumatoid arthritis and systemic lupus erythematosus [40,43]. AGP also functions as an immunomodulator [44]. It can induce cytokine release from mononuclear cells during an inflammatory response and can act in an anti-inflammatory manner by inhibiting the production of superoxide by neutrophils [45,46]. Therefore, the increase of AGP during SD is probably related to its anti-inflammatory and antioxidant functions.

Hp is an acute-phase protein that scavenges hemoglobin in the event of intravascular or extravascular hemolysis. In humans, this protein exists in three main phenotypes, Hp1-1, Hp2-2, and Hp2-1. Its genotype is reported to be associated with coronary artery disease and diabetes [47,48]. Hp was originally isolated from the liver as part of the acute-phase response to inflammation [49,50]. Accumulated data on the protein's function has established its strong association with diseases that have inflammatory etiology [51]. Hp has also been reported to play a proactive antioxidant role in the body [52]. Hp can also have an adipogenic effect by signaling the recruitment of inflammatory cells that contribute to chronic obesity-associated inflammation [53]. Therefore, an increase in Hp expression should have the same antioxidant function as TTR. However, in contrast to the effect of 60 and 100 msw SD on TTR expression level, 200 msw SD only increased Hp expression; therefore, longer exposure to hyperbaric hyperoxic conditions is likely required to activate the increased antioxidant functions of Hp.

Hyperbaric hyperoxia, such as that experienced during SD, is due to the increased partial pressure of oxygen and is generally believed to cause oxidative stress through the production of free radicals. However, there have been conflicting reports that showed that hyperbaric oxygen does not necessarily induce severe oxidative stress [54,55]. The beneficial aspects of hyperbaric oxygen therapy are well known, but the role of hyperbaric oxygen in free radical production remains to be elucidated. Additionally, hypobaric hypoxic conditions, especially when coupled with strenuous exercise, result in severe oxidative stress through several mechanisms. The limited availability of oxygen for oxidative reduction plays a major role in hypobaric hypoxic status [27], resulting in the accumulation of reactive oxygen species.

Proteomic analysis was first employed in the research of hypobaric hypoxia in 2013 [26]. Based on 2D DIGE combined with Matrix-assisted laser desorption/ionization/time-of-flight/time-of-flight (MALDI-TOF/TOF) spectrometry analysis, Padhy et al. [30] found seven proteins up-regulated in hypoxia-tolerant rats and apolipoprotein A-1 (APOA1) up-regulated in hypoxia-susceptible rats. They suggested that the proteins up-regulated in hypoxia work as antioxidants and could be putative biomarkers for the screening of altitude adaptation. The same group utilized the same proteomic analysis [29] to study human samples taken from control groups and high-altitude natives. They confirmed the up-regulation of the same seven proteins, including TTR and Hp beta chain, and the down-regulation of five proteins. They summarized that these up- and down-regulated proteins are well-known inflammatory inhibitors and play a positive anti-inflammatory role during hypobaric hypoxia. Later, they investigated blood samples from rats exposed to hypobaric hypoxia equivalent to 7620 m in a hypobaric chamber, to identify novel plasma proteins [30]. They identified 25 differently expressed proteins and confirmed eight proteins by western blotting and 2D DIGE followed by MALDI-TOF/TOF. They suggested that these proteins would be involved in cellular defense mechanisms for anti-inflammatory and antioxidant activity, and pointed out their potential utility as biological markers of hypobaric hypoxia. Transient up-regulation of TTR detected in humans under hypobaric hypoxia, as seen in saturation divers under hyperbaric hyperoxia, is also most likely an indication of the antioxidant and anti-inflammatory functions of TTR. It is believed that hyperbaric hyperoxia contrasts with hypobaric hypoxia, but the accumulation of abundant antioxidants seems to be a common phenomenon in both situations [27,28,29,30], and may be recognized as a unique coincidence. Obviously, the underlying mechanisms proposed to adapt to both extreme environments are quite different.

During the acute phase of infection, there is an increase in plasma acute phase proteins including Hp, AGP, serum amyloid A, and Pig major acute phase protein, accompanied by a decrease in the plasma level of TTR [56]. In the current study, high plasma levels of TTR were maintained during the 200 msw condition; however, a slight decrease in TTR expression was accompanied by high expression of AGP. When analyzing functional protein interactions using STRING (string-db.org), no direct interactions among TTR, AGP, and Hp were detected. Predicted functional partners of TTR and Hp include only one common protein, APOA1, which is also known to be an acute phase protein. Thus, TTR and Hp may function cooperatively via APOA1. Furthermore, the interaction between TTR and APOA1 was previously reported in a parasitic infectious disease [57]

Since the three proteins identified in this study have been previously shown to act as antioxidants, we speculate that in this study, their coordinated activity is an example of their biological effects as antioxidants under hyperbaric hyperoxia. Antioxidants are known to remove free radicals, most of which are chemically reactive species that contain oxygen. Free radicals are continuously formed as natural byproducts of the physiological metabolism of oxygen, and have important roles in redox cell signaling and homeostasis [58].

High pressure nervous syndrome (HPNS) is one of the important physiological manifestations resulting from deep SD. HPNS becomes clinically apparent with the depth and gradually reduces when the depth stabilizes, and disappears with ascend to the surface [9,59]. HPNS was first described as helium tremors in 1965 [59] and is a mysterious phenomenon thought to target the central nervous system. Symptoms of HPNS include tremors, somnolence, EEG changes, nausea, dizziness, and decreased mental performance [1,59].

To date, serious investigation has been conducted for the mechanisms underlying HPNS, mostly from electrophysiological stand points [59], including analysis of neurotransmission and membrane properties when the central nervous system is under pressure [59]. However, recent advancement in free radical research may show the link between the clinical appearance of HPNS and the accumulation of free radicals [60,61]. Therefore, we speculate that hyperbaric hyperoxia during SD might cause excessive production of free radicals that are beyond the capacity of the antioxidants including TTR, AGP and Hp, and thus, clinical manifestations of HPNS may occur. In the current study during 200 msw SD, protein expression of TTR increased with depth (average 1.21 at 100 msw and 2.21 17 h after arriving at 200 msw) and rapidly decreased after decompression (average 1.11 at 100 msw). In addition to this peculiar change in TTR expression level with the phase of diving, the excessive oxidation identified for TTR and AGP might support our hypothesis.

Traditionally, HPNS is known to become clinically apparent at a depth of approximately 150 msw [47, 48], but Thom et al. reported that hyperbaric helium increased the production of free oxygen radicals at as low as 2.8 ATA, which is equivalent to 18 msw (60 feet sea water) [62]. Therefore, the increased expression of TTR, AGP and Hp at the low depth of 60 msw SD is possible. However, we do not know why the expression of TTR (average 2.30) at 60 msw and 100 msw SD is slightly higher than that at 200 msw SD (2.18). It is possible that the slightly faster compression speed used for 60 msw and 100 msw SD may affect the protein expression.

Henschke et al. [63] investigated the proteomic expression differences of *ex vivo* fetal rabbit lungs under normoxia (21% O_2_, 5% CO_2_) and hyperoxia (95% O_2_, 5% CO_2_) using two dimensional gel-electrophoresis combined with MALDI-TOF MS and MALDI-MS/MS. Briefly, they identified 12 proteins including vimentin, annexin I, inorganic pyrophosphatase, prohibitin, an N-terminal fragment of ATP synthase, and heat shock protein 27. However, the three proteins identified in this study were not found by Henschke et al. This difference suggests that hyperoxia alone is not sufficient to increase TTR, AGP and Hp levels, but simultaneous high pressure is required. Obviously, there are caveats to our speculation, such as species differences (rabbit vs. human) and experimental protocol differences (*ex vivo* vs. *in vivo*). Still it is interesting that high pressure with hyperoxia, but not pure hyperoxia, is crucial for the up-regulation of TTR, AGP, and Hp.

Recently, Lautridou et al. applied the proteomics based approach for rat blood samples showing decompression sickness (DCS) after simulated air diving [64]. Comparing the DCS group and the non-DCS group, they identified three proteins (APOA1, serine protease inhibitor A3K, and alpha-1-antiproteinase) that increased in the DCS group, but displaying only slight changes. Interestingly, by contrast, TTR level drastically decreased in the DCS group. Our current data, taken together with the non-DCS group, show that TTR increases during the bottom phase of SD and returns to its pre-diving value; however, once DCS occurs, serum TTR level decreases. Therefore, TTR might be a possible plasma biomarker for DCS diagnosis and for hyperbaric exposure.

This identification has a strong impact on the diving medicine field as there is no universally accepted diagnostic criterion for DCS despite over 100 years of experience [65]. The measurement of TTR might predict the occurrence of delayed manifestation DCS (free of symptoms immediately after surfacing), mostly within 12 h after surfacing. Mild symptoms such as paresthesia, tingling, headache, etc. are frequently encountered in out-patient clinic for diving injury, but the symptoms are difficult to interpret because they may be due to other causes besides DCS. As Eftedal et al. described [66], we agree that TTR as a proposed DCS biomarker may offer an insight into DCS etiology.

In addition, they described that the decrease in TTR level in DCS rats was because of TTR working as a negative acute phase protein and suggested that hyperbaric conditions could trigger an acute inflammation leading to DCS. However, during exposure to hyperbaric hyperoxia, as seen in SD, excess TTR level needs to be normalized when returning to the surface. With prolonged durations, overexpression of TTR might accumulate in human tissue as seen in several pathologies including familial amyloid polyneuropathy [67], Alzheimer disease [38,39,68], and cardiomyopathy [69]. Accumulation of harmful amyloids detected by dot blot using anti-mouse monoclonal antibody against amyloidβE22P (11A1, Immuno-Biological Laboratory, Japan) may, in part, support our speculation (data not shown). Although we suggested that TTR might be a possible plasma biomarker for DCS diagnosis, there is obvious limitation in our study. Our study lacks the comparison between those who show DCS during SD and those who do not. However, as far as we continue SD experiments and training according to our current system, the occurrence rate of DCS during SD is almost zero. Therefore, it is not easy to have the samples from DCS patients during SD. We may have to increase the sample volume either by increasing the time of blood draw during SD or by increasing the number of participants with the current experimental protocol. To study further details of TTR under a hyperbaric environment and in DCS patients, we may choose DCS patients frequently involved in recreational diving. Analyzing blood samples of patients receiving regular hyperbaric oxygen treatments compressed to 60 feet sea water (2.8 ATA) might be another strategy for future investigation.

In summary, our proteomic analysis revealed for the first time that the antioxidant and anti-inflammatory proteins TTR, AGP, and Hp were up-regulated during SD. Due to the coordinated movement of TTR, AGP, and Hp with diving phase and the relationship with expected free radical production, biological manifestation of these three proteins might be related to HPNS. The method used in this study is not sensitive enough to detect the differential expression of plasma proteins during SD, mainly because albumin and immunoglobulins were included in the samples applied in 2D DIGE. As albumin and immunoglobulins are known to have a key role in overall antioxidant defense mechanism, we could not exclude the involvement of these proteins in the mechanism of adaptation to hyperbaric conditions before this study [70]. Our study showed that albumin and immunoglobulins did not vary under conditions of SD, and a more sensitive proteomic analysis of blood samples excluding these proteins is needed. The mechanism how hyperbaric hyperoxia affects the up-regulation of TTR, AGP, and Hp should be elucidated among various environmental factors of SD such as a claustrophobic environment and high humidity.
